# Bayesian Multi-objective Hyperparameter Optimization for Accurate, Fast, and Efficient Neural Network Accelerator Design

**DOI:** 10.3389/fnins.2020.00667

**Published:** 2020-07-21

**Authors:** Maryam Parsa, John P. Mitchell, Catherine D. Schuman, Robert M. Patton, Thomas E. Potok, Kaushik Roy

**Affiliations:** ^1^Department of Electrical and Computer Engineering, Purdue University, West Lafayette, IN, United States; ^2^Computational Data Analytics, Oak Ridge National Laboratory, Oak Ridge, IN, United States

**Keywords:** multi-objective hyperparameter optimization, Bayesian optimization, neuromorphic computing, spiking neural networks, accurate and energy-efficient machine learning

## Abstract

In resource-constrained environments, such as low-power edge devices and smart sensors, deploying a fast, compact, and accurate intelligent system with minimum energy is indispensable. Embedding intelligence can be achieved using neural networks on neuromorphic hardware. Designing such networks would require determining several inherent hyperparameters. A key challenge is to find the optimum set of hyperparameters that might belong to the input/output encoding modules, the neural network itself, the application, or the underlying hardware. In this work, we present a hierarchical pseudo agent-based multi-objective Bayesian hyperparameter optimization framework (both software and hardware) that not only maximizes the performance of the network, but also minimizes the energy and area requirements of the corresponding neuromorphic hardware. We validate performance of our approach (in terms of accuracy and computation speed) on several control and classification applications on digital and mixed-signal (memristor-based) neural accelerators. We show that the optimum set of hyperparameters might drastically improve the performance of one application (i.e., 52–71% for Pole-Balance), while having minimum effect on another (i.e., 50–53% for RoboNav). In addition, we demonstrate resiliency of different input/output encoding, training neural network, or the underlying accelerator modules in a neuromorphic system to the changes of the hyperparameters.

## 1. Introduction

Neuromorphic systems promise a novel alternative to the standard von-Neumann architectures that are computationally expensive for analyzing big data, and are not efficient for learning and inference. This novel generation of computing aims at “mimicking” the human brain based on deploying neural networks on event-driven hardware architectures. A key bottleneck in designing such brain-inspired architectures is the complexity of co-optimizing the algorithm's speed and accuracy along with the hardware's performance and energy efficiency. This complexity stems from numerous intrinsic hyperparameters in both software and hardware that need to be optimized for an optimum design.

In this work we propose a novel optimization framework built upon agent-based modeling and hierarchical Bayesian optimization techniques to obtain the optimum set of hyperparameters for neuromorphic system design. Bayesian optimization is a powerful tool for finding the optimal point of objective functions that are unknown and expensive to evaluate (Shahriari et al., 2015). However, for problems with more than one objective function Bayesian-only techniques are mathematically complex, and suffer from high dimensionality limitations in parameter-heavy models (Dai et al., 2019). Other approaches such as Neural Architecture Search (NAS, Zoph et al., 2018) also require massive computational resources. These factors were the driving forces to search for alternative algorithms to find the optimal set of hyperparameters.

Our proposed approach, Hierarchical Pseudo Agent-based Bayesian Optimization (Hierarchical-PABO), is built upon using a supervisor agent correlating the results of isolated Bayesian estimations for each of the objective functions. The agent creates an extra set of Bayesian estimator focusing only on finding the Pareto frontier. The hierarchy of Bayesian optimizers enables predicting the Pareto frontier for complex problems regardless of the number of objective functions. In comparison with our previous works in (Parsa et al., 2019a,b), H-PABO is a general framework that covers both PABO (Parsa et al., 2019a) and single-objective Bayesian optimization (Parsa et al., 2019b) under its umbrella. In Parsa et al. (2019a), we introduced PABO, which was the initial phase toward designing Hierarchical PABO. PABO has no hierarchy of Bayesian estimators, and the supervisor agent decides the search direction in favor of the Pareto region, without any Bayesian estimator. By turning off the extra set of Bayesian estimators that are used to predict the Pareto frontier, H-PABO reduces to PABO. In Parsa et al. (2019b), we used a single objective hyperparameter Bayesian optimization to optimize performance of spiking neuromorphic systems in terms of neural network's accuracy. We showed how critical it is to use hyperparameter optimization techniques for designing any neuromorphic computing framework and how Bayesian approaches can help in this regard. H-PABO reduces to a single objective hyperparameter optimization problems when the number of objectives functions are fixed to one.

We tested Hierarchical-PABO on both artificial neural networks and spiking neural networks. For artificial neural networks, we validated our approach using AlexNet (Krizhevsky et al., 2012) and VGG19 (Simonyan and Zisserman, 2014) on a Programmable Ultra-Efficient Memristor-based Accelerator (PUMA, Ankit et al., 2019). For spiking neuromorphic systems, we considered several control and classification tasks such as the canonical pole balancing (Gomez et al., 2006), autonomous robotic navigation (Mitchell et al., 2017), satellite radio signal classification (Reynolds et al., 2018), and Iris dataset classification (Dua and Graff, 2017) on both digital and mixed-signal memristor-based accelerators as the underlying hardware (Chakma et al., 2017; Mitchell et al., 2018; Plank et al., 2018). Hierarchical-PABO predicts the Pareto frontier for a three-objective (network performance, the accelerator's energy efficiency, and area) optimization with relatively few evaluations. Compared to the state-of-the-art methods, our framework is faster by at least an order of magnitude and as effective, if not more, in finding an optimal solution. Further, the speed and accuracy of the framework enables designers to perform sensitivity analyses on hyperparameters to determine the resiliency of the system to the changes of the hyperparameters.

### 1.1. Background and Related Work

In the era of the exigent need to design energy efficient neuromorphic systems for resource-constrained environments such as mobile edge devices, several approaches have been proposed in the literature to reduce the massive energy requirement of these systems. For artificial neural networks (ANNs), these techniques span from simplifying models, such as pruning and quantization (Han et al., 2015; Wen et al., 2016; Yang et al., 2018; Zoph et al., 2018), to designing energy efficient architectures (Jin et al., 2014; Panda et al., 2016; Parsa et al., 2017; Wang et al., 2017), and neural architecture search (Zoph et al., 2018). In spiking neuromorphic domain, these include different training algorithms such as Schuman et al. (2016), Bohnstingl et al. (2019) based on evolutionary optimization, Esser et al. (2015, 2016) on modified backpropagation techniques, Severa et al. (2019) as binary communication, and Rathi et al. (2020) as a hybrid approach and then deploying these on neuromorphic hardware such as Schmitt et al. (2017) and Koo et al. (2020). In this section, we briefly introduce each of these methods and continue with the added complexity of co-designing hardware and software for artificial neural networks and spiking neuromorphic systems. We then present the contribution of our work (Hierarchical-PABO) and how we fill the existing gap in a generic approach of co-designing hardware and software in the literature.

To reduce the energy requirement of neural network architectures, model simplification techniques proposed by Han et al. (2015), and continued with Wen et al. (2016), Zoph et al. (2018), and Yang et al. (2018). Each of these techniques focus on simplifying the neural network with different approaches of pruning, quantization, learning the connections, and leveraging sparsity. Designing energy-efficient architectures are also well-studied in the literature with flattened Convolutional Neural Network (CNN) (Jin et al., 2014), factorized CNN (Wang et al., 2017), conditional CNN (Panda et al., 2016, 2017), and staged-conditional CNN (Parsa et al., 2017). More recently, compact structures such as MobileNets (Howard et al., 2017) and ShuffleNet (Zhang et al., 2018) are also introduced and are specifically designed for mobile devices. Although both approaches of model simplification and efficient architecture design demonstrate promising results in reducing the energy requirements of neural networks, they do not necessarily yield to the optimum designs for energy efficient accelerators. This is mainly due to the fact that they only locally search the space. In addition, layers with more parameters do not necessarily consume more energy (Yang et al., 2017; Dai et al., 2019). Various techniques proposed for training spiking neural networks with different underlying hardware, are vital steps toward efficient neuromorphic computing for edge devices; however, each of these approaches require several hyperparameters and their optimum performance depend on prior knowledge on how to set these hyperparameters. In Parsa et al. (2020), we showed that an optimum set of hyperparameters drastically increases the neuromorphic system performance.

There is a very rich literature on hyperparameter optimization and neural architecture search (NAS) techniques. Search for the optimum set of hyperparameters studied by Genetic CNN (Xie and Yuille, 2017), metaQNN (Baker et al., 2017), and SMBO (Liu C. et al., 2018). These techniques are built upon using Genetic algorithms or Bayesian optimizations. NAS was started by Google Brain (Zoph et al., 2018) to find an optimal neural architecture by searching for architectural building blocks on a small dataset and then transferring the block to larger ones. NAS was a starting point for a series of NAS-based approaches in recent years (Liu C. et al., 2018; Liu H. et al., 2018; Pham et al., 2018). All of these works were proposed to design a neural network with optimum performance, regardless of the energy requirement of the underlying neural accelerator.

Hardware-aware neural architecture designs can be categorized in three domains of multi-layer co-optimization (Reagen et al., 2016), hardware-aware NAS (Cai et al., 2018; Tan et al., 2019; Wu et al., 2019), and Bayesian-based hyperparameter optimization (Reagen et al., 2017; Marculescu et al., 2018; Stamoulis et al., 2018). Each one of these approaches have their pros and cons. While defining an optimum neural architecture with energy-efficient hardware in mind, the multi-layer co-optimization approach cannot easily be extended to generic platforms. Hardware-aware NAS techniques are time consuming and require substantial resources, and Bayesian-based methods are not well-suited for parameter-heavy models Dai et al. (2019). In Hierarchical-PABO, we propose a novel hardware-aware approach with minimum mathematical complexity. This framework is based on hierarchical Bayesian optimization and agent-based modeling. Using a set of Bayesian estimators in different levels and correlating them using a supervisor agent, we overcome the drawbacks of exclusive Bayesian approaches available in the literature.

### 1.2. Main Contributions

We made the following contributions:

1. ***A novel optimization framework based on hierarchical Bayesian optimization and agent-based modeling, suitable for both artificial neural networks and spiking neuromorphic systems***. With simple yet effective underlying mathematics, Hierarchical-PABO estimates the Pareto region for multi-objective hyperparameter optimization problems with few evaluations.
2. ***One of the first techniques in the literature for co-designing software-hardware that is not limited to the number of objectives to optimize (network performance, energy consumption, size, speed of inference, etc.)***. Based on our knowledge, our proposed technique is one of the first techniques in the literature that simplifies the mathematical complexity of exclusive Bayesian approaches for multi-objective optimization. We do this by adding a supervisor agent and performing Bayesian optimization in different levels. This paves the way to effectively optimize more than two objective functions.
3. ***Generic framework extendable to various artificial and spiking neural networks and the underlying digital, analog, or mixed-signal accelerators***. We tested our framework on several classification and control applications on digital and mixed-signal accelerators and were able to estimate the Pareto frontier regardless of the size of the search space.
4. ***Superior performance in terms of accuracy and computational speed compared to the state-of-the-art Genetic Algorithm (GA) optimization approach*** (in scenarios where GA-based optimizations were available for comparison, Deb et al., 2002). Please see Parsa et al. (2019a) for details of this contribution.

## 2. Methodology and Experimental Setup

In order to systematically take the human knowledge out of the loop in selecting the optimum set of hyperparameters for a neuromorphic system (and in general any artificial intelligence-based computing system), we chose Bayesian optimization as the core of our approach. In this section, we first overview the basic mathematics of Bayesian modeling and justify the use of this technique in our proposed Hierarchical Pseudo Agent-based Bayesian Optimization (Hierarchical-PABO) framework, and then present the experimental setup for this approach.

### 2.1. An Introduction to Bayesian Optimization

Bayesian optimization is a powerful tool for finding the optimum point of objective functions that are unknown and expensive to evaluate (Brochu et al., 2010). The problem of finding a global optimizer for an unknown objective function is formulated in Equation (1).

(1) $$\begin{array}{l} x^{*}=\underset{x\in X}{\mathrm{argmax}}f\left(x\right) \end{array}$$

where *X* is the entire design space, and *f* is the black-box objective function without simple closed form. As summarized by Shahriari et al. (2015), in a sequential manner, we search for the best location *x*_*n*+1_ to observe *y*_*n*+1_ point in order to estimate *f*. After *N* iterations, the algorithm suggests the best estimation of the black-box function *f*. This sequential approach is based on building a prior estimation over possible objective functions, and then iteratively re-estimating the prior model using the observations from updating the Bayesian posterior model. The posterior representations are the updated knowledge on the objective function we are trying to optimize. We explore the search space by leveraging the inherent uncertainty of the posterior model and mathematically introducing a surrogate model, called the acquisition function α_*n*_. The maximum point of this function is the next candidate point to observe (*x*_*n*+1_) and guides the search direction toward the true representation of the objective function. The efficiency of Bayesian approach to estimate the global optimizer for the expensive black-box function with fewer evaluations lies on the ability of Bayesian technique to learn from prior belief on the problem and direct the observations by trading off exploration and exploitation of the design space.

**Figure 1:**
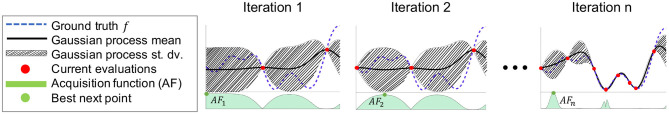
Summary of single objective Bayesian optimization. Reproduced with permission from Parsa et al. (2019a).

In the context of neuromorphic computing, *x* is the system's hyperparameters such as inherent hyperparameters for different input/output encoding schemes, or population size or optimizer choice for various training techniques. Hardware-specific hyperparameters are also another choice for parameter *x*. Function *f* is the black-box objective function, such as accuracy of the network, energy or area requirements of the system, and speed of inference, for stochastic observations of *y*. A summary of the Bayesian approach is illustrated in the Figure 1. See Brochu et al., 2010; Bergstra et al., 2011; Eggensperger et al., 2013 for detailed tutorials.

In Figure 1, we are estimating an unknown objective function, ground truth *f*. We only have two observations (likelihood model) in iteration one (red dots). We first build our prior distribution (current belief) based on these observations using Gaussian processes. The Gaussian distribution is shown with mean and standard deviation, solid black line, and highlighted dashed area, respectively. A surrogate model, acquisition function, is estimated for this posterior distribution, which is shown as the highlighted green function. The maximum point of the acquisition function (green dot) is the best next point to observe in the next iteration. As the new points are added to the observations in different iterations, the standard deviations, and therefore the uncertainty of estimating the ground truth function, is reduced. Each observation requires evaluating an unknown, expensive objective function. The ability of the Bayesian technique in predicting this function (ground truth in Figure 1) with few evaluations, speeds up the process of finding the optimum set of hyperparameters with minimum computational resources.

For configuring the Gaussian process, the covariance function is a positive definite kernel that specifies the similarity between points of observations. There are different methods to estimate this kernel function based on the smoothness, noise level and periodicity of the ground truth. In our experimental setup, we selected the Matern kernel function with smoothness value of 1.5. This particular kernel is selected due to the intrinsic stochastic nature, and noise level of our problem. Once we estimate the posterior distribution based on the likelihood model and the prior distribution, we build an acquisition function to guide the search direction. This acquisition function defines whether to search the space where the uncertainty is high (explore) or sample at locations where the model predicts high objectives (exploit). There are different methods to calculate this surrogate model (Kushner, 1964; Lai and Robbins, 1985; Jones et al., 1998; Jones, 2001; Brochu et al., 2010; Bull, 2011; Agrawal and Goyal, 2013; Hernández-Lobato et al., 2014). The choice of the method to use directly impacts the speed of convergence to the ground truth in Bayesian search. We chose expected improvement approach for the acquisition function. This selection does not impact the effectiveness or performance of our approach; rather, it only impacts the speed of searching the hyperparameter space and avoid trapping in local minima. More details in selecting kernel or acquisition function can be found in Shahriari et al. (2015).

### 2.2. Hierarchical-PABO

Hierarchical-PABO (Hierarchical Pseudo Agent-based Bayesian Optimization) is an ultra-efficient Bayesian-based optimization framework to find an optimum set of hyperparameters for designing an accurate neural network while minimizing energy consumption and area requirement of the underlying hardware.

**Figure 2:**
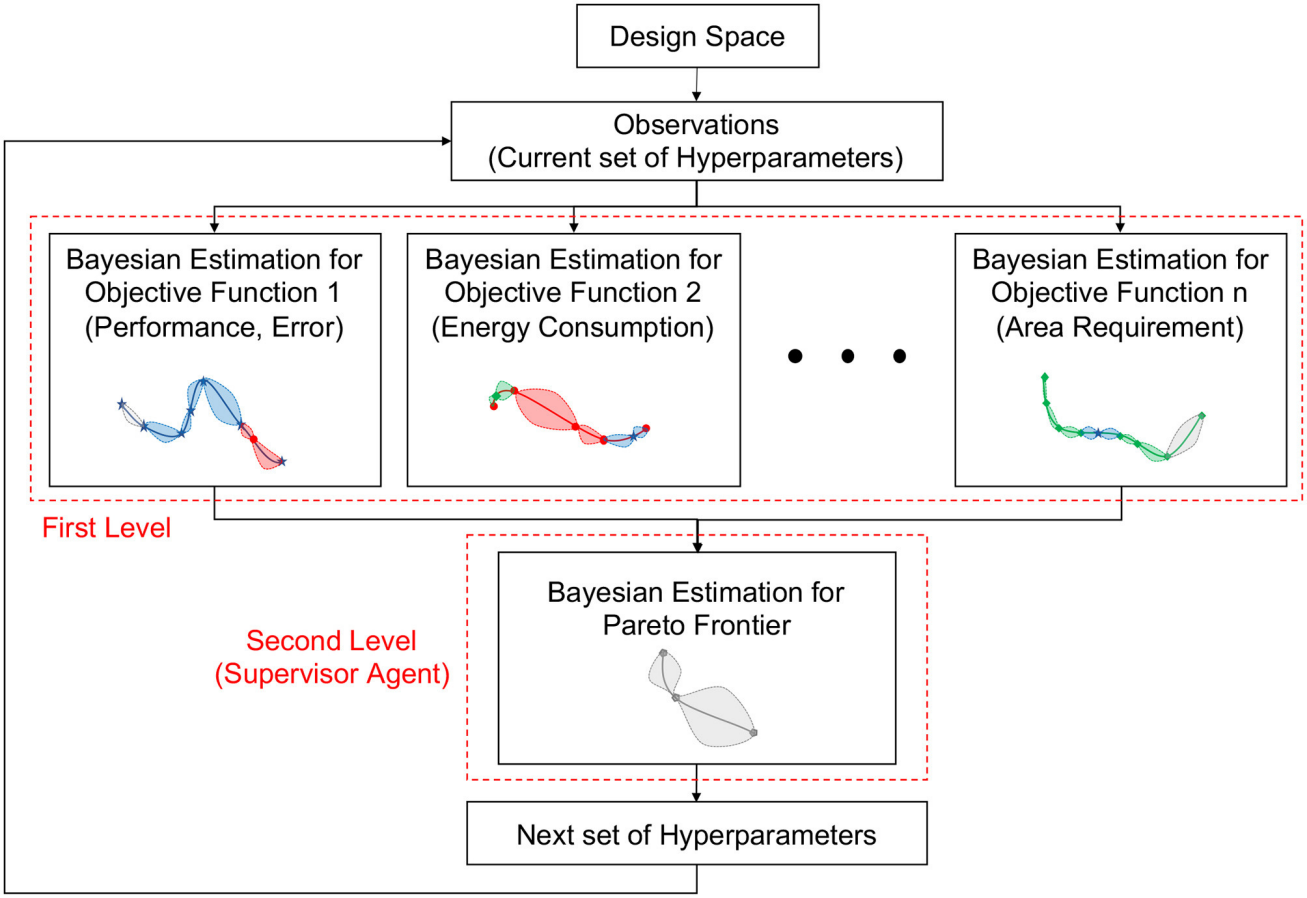
Hierarchical-PABO framework.

Figure 2 summarizes the Hierarchical-PABO framework. We randomly select two hyperparameter (HP) combinations from the design space. In the first level, these current observations are used to build Bayesian estimation posterior distributions for each objective function separately. We then define the acquisition function for each posterior model. The optimum point of these acquisition functions are the best next point (HP combination) to evaluate for their corresponding objective function. In the second level, the supervisor agent level, the process starts with all current observations (set of HP combinations) and the candidate HP combination that led to the optimum value of the acquisition functions in the previous iteration. For these observations, we estimate an intermediate Pareto frontier function using a Gaussian distribution. This is calculated based on the observation points (on the Pareto front set), as well as a score calculated based on L1-norm of these points after being normalized. Therefore, a corresponding surrogate model (acquisition function) for this Gaussian distribution explores and exploits the search space with the goal of estimating the current intermediate Pareto function. The next best observation for this Pareto is then added to the observations for each Bayesian estimator. With this technique, we force the Bayesian approach to add extra observations that help in minimizing the current intermediate Pareto function. This function is updated iteratively and moved toward the actual Pareto region of the problem.

In Hierarchical-PABO, the Pareto Bayesian estimator in the second level plays a vital role in correlating the Bayesian estimators for each objective function in the first level. However, to speed up the search process, the supervisor agent might turn off this Pareto Bayesian estimator. If this extra Bayesian estimator is turned off, the supervisor agent takes HP combinations taken from optimum point of the acquisition function for each objective and only allow those that are in favor of moving toward the Pareto region. Please see the Supplementary Material for Hierarchical-PABO pseudo-code.

### 2.3. Experimental Setup

**Figure 3:**
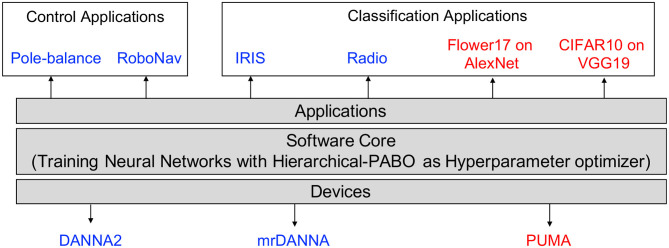
Summary of the experimental setup (ANN and SNN).

An overview of our experimental setup is shown in Figure 3. We test Hierarchical-PABO on several devices for various control and classification tasks. For experiments on Artificial Neural Networks (ANNs), we select PUMA (Ankit et al., 2019) as the underlying hardware with two different deep neural network architectures, AlexNet (Krizhevsky et al., 2012) and VGG19 (Simonyan and Zisserman, 2014) on Flower17 (Nilsback and Zisserman, 2006), and CIFAR10 (Krizhevsky, 2009) image classification dataset. For Spiking Neural Networks (SNNs), we consider both digital and mixed-signal hardware; DANNA2 (Mitchell et al., 2018), and mrDANNA (Chakma et al., 2017), respectively. Additionally, we select Pole-balance (Wieland, 1991; Gomez et al., 2006), and RoboNav (Mitchell et al., 2017) for experiments on control applications, and IRIS (Dua and Graff, 2017), and Radio (Reynolds et al., 2018) dataset for classification applications. In Figure 3, the experimental setup for ANN is shown in red, and for SNN in blue.

In SNN domain, we utilize a modified version of the TENNLab neuromorphic software framework (Plank et al., 2017, 2018). This platform enables studying different applications and evaluating them on several neuromorphic processor implementations. This capability is well-suited for the purpose of our hyperparameter multi-objective optimization as it allows switching applications and devices within the framework without the need to change the software. We modify the TENNLab framework by adding Hierarchical-PABO to its primary underlying learning algorithm, which is Evolutionary Optimization for Neuromorphic Systems, EONS (Schuman et al., 2016). EONS is an evolutionary approach for designing the network topology and parameters of an SNN for a given application and neuromorphic hardware implementation. This evolutionary algorithm follows the same steps as a traditional evolutionary approach. That is, EONS begins with a population of potential solutions and evaluates each of those solutions on the problem at hand (running the potential network solution on the application on the hardware or a simulation of the hardware) to get a fitness score for each solution. Then, EONS uses the fitness scores to perform selection (preferentially selecting better performing networks to serve as parents) and reproduction (to produce children networks from the parents). Reproduction includes both crossover operations (taking components from two networks to assemble one or more children), mutation operations (small-scale changes such as parameter updates or adding or deleting a neuron or synapse), and duplication. Details of the experimental setup for both ANN and SNN are described in this section.

#### 2.3.1. Experimental Setup for ANN

For experiments in ANN domain, to speed up the search for the optimum hyperparameter, we turn off the extra Bayesian estimator block in the supervisor agent. In this case, the supervisor agent only correlates the results of the isolated Bayesian estimations of each objective function, and decides on the best hyperparameter combination for the next iteration based on the ones that might belong to the Pareto frontier. Details of the Hierarchical-PABO when the extra Bayesian estimator in supervisor agent is turned off is given in Parsa et al. (2019a).

As discussed in Parsa et al. (2019a), the underlying hardware we select for our ANN experimental setup is a programmable ultra-efficient memristor-based accelerator called PUMA, proposed by Ankit et al. (2019). This spatial general-purpose architecture is based on hybrid CMOS-memristor technology that enables mapping machine learning applications using on-chip memory only. Analog memristor crossbars, functional units, and instruction execution pipelines are the building blocks of PUMA's core. Multiple cores create tiles via a shared memory. PUMA's nodes are several tiles connected through an on-chip network. For large-scale executions, PUMA nodes are linked with a chip-to-chip interconnect.

To calculate energy consumption of PUMA, we use an abstract energy consumption model of the memristor crossbars only. This enables evaluating the impact of hyperparameters on the energy usage of PUMA, while isolating the benefits of micro-architectural design. We expect lower energy usage with less number of crossbars. Details of calculating the energy consumption of PUMA is given in Equation (2).

(2) $$\begin{array}{l} Total\;Energy=[\sum_{i}(d_{i}\times d_{i}\times \left\lceil \frac{nc_{i}\times k_{i}\times k_{i}}{xs}\right\rceil \times \left\lceil \frac{nc_{i+1}}{xs}\right\rceil )\\ \;+\sum_{i}(\left\lceil \frac{nf_{i}}{xs}\right\rceil \times \left\lceil \frac{nf_{i+1}}{xs}\right\rceil )]\times epx \end{array}$$

In Equation (2), the total energy consumption is the summation of number of crossbars needed for all convolution and fully connected layers multiplied by the energy per matrix vector multiplication operation (*epx*). In PUMA's memristive crossbar accelerator, *epx* is ≃44 nJ for a 16-bit (inputs and weights) crossbar operation with crossbar size (*xs*) of 128 × 128. For the *i*_*th*_ convolution layer, *d*_*i*_ is the dimension of the output, *nc*_*i*_ is the number of input features, and *k*_*i*_ is the kernel size. The dimension of the output in the convolution layer is for the inherent weight-sharing property of these layers. For the *i*_*th*_ fully connected layer, *nf*_*i*_ is the number of input features.

In the ANN's experimental setup, we used AlexNet (Krizhevsky et al., 2012) and VGG19 (Simonyan and Zisserman, 2014) for the deep neural network architectures. For details on the structures of AlexNet and VGG19 please refer to the Supplementary Material. We performed several case studies for different types of hyperparameters, including the number of layers, kernel sizes, number of features to extract in each layer, and also the values for learning rate, momentum, and dropout. The details of the our proposed hyperparameter optimization technique on ANN results are given in Parsa et al. (2019a).

#### 2.3.2. Experimental Setup for SNN

As mentioned in section 2.2, based on the complexity of the problem, the supervisor agent decides to keep the extra Bayesian estimator block on or off. In SNN domain, this block is turned on which is well-suited for the hyperparameter optimization of spiking neuromorphic systems. In these systems, the intrinsic HPs in different building blocks of these systems are so critical in the final performance of the system that an additional Bayesian optimizer is needed to find the optimum set of HPs.

The summary of the applications and neuromorphic processors we select for SNN experimental setup is shown in Figure 3. For the applications, we tested Hierarchical-PABO on both control and classification tasks. Pole-balance (Wieland, 1991; Gomez et al., 2006), and RoboNav (Mitchell et al., 2017) were the two selected control applications. Pole-balance is a control benchmark in engineering which involves a pole connected to a cart through a joint that allows single axis movement. The goal of this control application is to keep the pole from falling by moving the cart either direction. RoboNav is an autonomous navigation system for robotic applications and is meant to be deployed on a specific robot (Mitchell et al., 2017). We also used the Iris (Dua and Graff, 2017) and Radio (Reynolds et al., 2018) datasets for classification tasks. The former is a multivariate dataset of 50 samples from each of three species of the Iris flower, and the latter is a satellite radio signal classification problem.

We use two different neuromorphic implementations that are already deployed in the TENNLab framework, a fully digital neuromorphic processor, DANNA2 (Mitchell et al., 2018), and a memristive mixed-signal neuromorphic processor, mrDANNA, (Chakma et al., 2017). DANNA2 is a fully digital programmable device with integrate-and-fire neurons and synapses, and mrDANNA is a mixed analog-digital programmable device with metal-oxide memristors. We use mrDANNA for the case studies where we would like to minimize energy requirement of the underlying neuromorphic hardware. Table 1 summarizes the energy estimate per spike for this neuromorphic device. mrDANNA is a synchronous neuromorphic architecture and is simulated in a discrete event simulation. Events in the simulation include accumulations, fires, and learning. The energy estimates for each event type are given in Table 1 and we track how many of each type of event occurs in the simulation and sum up the energies. If no event is occurring on a neuron or synapse in a clock cycle, that neuron or synapse is “idle,” but still performing some operations that contribute to idle cost. We use these energy estimates to estimate the overall energy cost of running on a particular application.

**Table 1:** Energy estimate per spike for mrDANNA.

	**Accumulation**	**Fire**	**Learning**	**Idle**
Neuron	9.81*pJ*	12.5*pJ*	-	7.2*pJ*
Synapse	1.45*pJ*	-	2.58*pJ*	0.07*pJ*

## 3. Results

To validate Hierarchical-PABO we consider different case studies, which are summarized in Table 2. Different applications (control and classification), architectures (AlexNet and VGG19 for ANN, and EONS for SNN), dataset (Flower17, CIFAR10, IRIS, Radio, and Pole-balance), and accelerators (PUMA, DANNA2, mrDANNA) are considered with different search space sizes. These different case studies are chosen to demonstrate our proposed generic hyperparameter optimization approach.

**Table 2:** Case studies for hierarchical-PABO.

**Case study**	**Domain**	**Application**	**Architecture**	**Dataset**	**Accelerator**	**Search space**	**Objective**
One	ANN	Classification	AlexNet	Flower17	PUMA	192	Accuracy, Energy
Two	ANN	Classification	AlexNet	Flower17	PUMA	288	Accuracy, Energy
Three	ANN	Classification	VGG19	CIFAR10	PUMA	3,072	Accuracy, Energy
Four	SNN	Control	EONS	Pole-Balance	DANNA2	240	Accuracy
Five	SNN	Control	EONS	Pole-Balance	DANNA2	54,432,000	Accuracy
Six	SNN	Classification	EONS	IRIS	mrDANNA	1,458	Accuracy, Energy, Size
Seven	SNN	Classification	EONS	Radio	mrDANNA	1,458	Accuracy, Energy, Size
Eight	SNN	Classification	EONS	IRIS	mrDANNA	35,460	Accuracy, Energy, Size
Nine	SNN	Classification	EONS	Radio	mrDANNA	35,460	Accuracy, Energy, Size

### 3.1. Results for ANN

Table 3 shows a summary of the selected ranges for the hyperparameters (HPs) for each ANN case study given in Table 2. All these cases are studied with PUMA as the underlying hardware. Case study one is designed with a small search space of size 192 HPs. We begin with the small search space size in order to estimate the actual Pareto frontier of the problem with a grid search technique and to compare the Hierarchical-PABO (H-PABO) result with other state-of-the-art approaches. Case study two is included to capture the effects of different types of HPs in the analysis, and case study three is a more realistic experiment with VGG19 as the chosen architecture on CIFAR10 dataset.

**Table 3:** Evaluated parameters for three different case studies for ANNs.

	**Case study one**	**Case study two**		**Case study three**
Dropout	0.4, 0.5	0.5	Dropout, Layer 1	0.3, 0.4
Learning Rate	0.001	0.001, 0.01	Learning Rate	0.01, 0.1
Momentum	0.85, 0.9, 0.95	-	Learning Rate Decay	1*e* − 6, 1*e* − 4
Optimizer	Momentum	Momentum, Adam	Weight Decay	0.0005, 0.05
# of FC Layers	2, 3	2, 3	Kernel Size, Layer 6	3, 5
# of Conv. Layers	4, 5	3, 4, 5	Kernel Size, Layer 7	3, 5
Kernel Size, Layer 1	5, 7	3, 5, 7	Kernel Size, Layer 8	3, 5
Kernel Size, Layer 2	3, 5	3, 5	Kernel Size, Layer 9	3, 5, 7
Kernel Size, Layer 3	3, 5		# of Features, Layer 1	64, 128
Kernel Size, Layer 4	3	3, 5	# of Features, Layer 2	128, 256
			# of Features, Layer 4	256, 512
Architecture	AlexNet	AlexNet		VGG19
Neural Accelerator	PUMA	PUMA		PUMA
Dataset	Flower17	Flower17		CIFAR10
Search Space	192	288		3072

**Figure 4:**
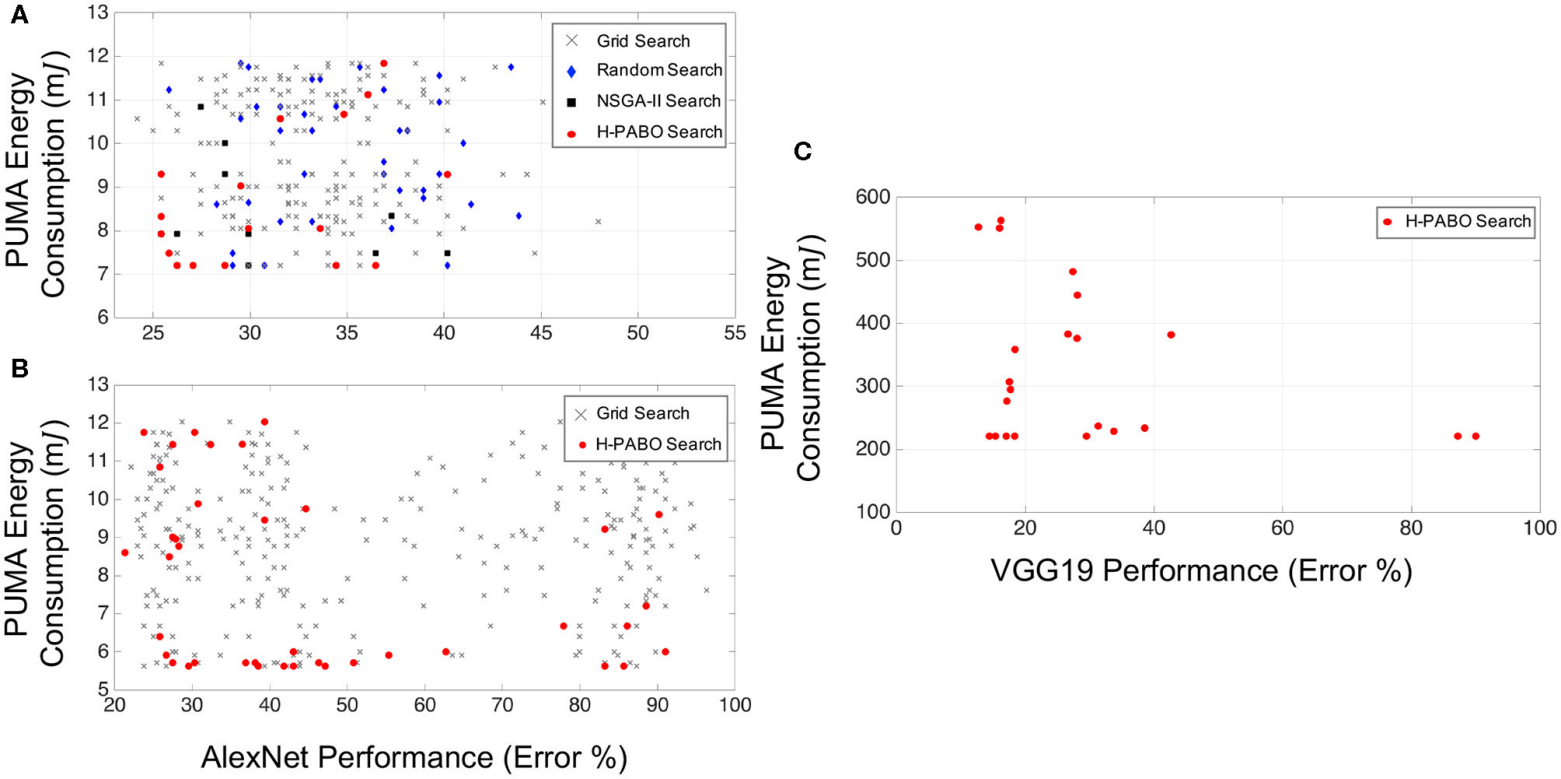
ANN results for multi-objective hyperparameter optimization of network performance and hardware energy requirement. **(A)** Case study one: HP search space = 192, Reproduced with permission from Parsa et al. (2019a). **(B)** Case study two: HP search space = 288. **(C)** Case study three: HP search space = 3,072, Reproduced with permission from Parsa et al. (2019a).

Figure 4 demonstrates results for different case studies. Each point in this figure corresponds to a set of HPs from the ranges given in Table 3. H-PABO search points are shown in red circles and are the selected HP combinations that lead to defining a Pareto frontier region. As already discussed in section 2.2, this selection is based on exploring and exploiting the search space. In all three case studies shown in Figure 4, the H-PABO search not only emphasizes on the Pareto region, but also explores the search space to avoid trapping in local minima.

In Figure 4A, H-PABO search points are compared to grid search (shown in gray crosses), random search (blue diamonds), and state-of-the-art NSGA-II (Deb et al., 2002) search (black squares). H-PABO predicts the actual Pareto frontier of the problem with only 17 evaluations (out of 192 possible HP combinations). This result outperforms other approaches not only in accuracy of predicting the Pareto frontier, but also in superior computational speed. The random search results are from 40 evaluations of HP combinations, and NSGA-II is based on a population size of 10 with a maximum generation of 50. In this case study, H-PABO is 92× faster than NSGA-II in predicting the actual Pareto frontier of the problem. An optimal design that belong to the Pareto frontier with 26% error and 7*mJ* PUMA energy consumption will lead to almost 40% decrease in energy consumption compared to a not-optimal design with 26% error and 12*mJ* energy consumption. For these two designs all hyperparameters such as dropout, learning rate, and optimizer type are similar, except number of fully connected layers, convolution layers, and two filter sizes. The optimal design has two fully connected layers, and four convolution layers with filter sizes 3 in the second and third layers. However, the not-optimal design has three fully connected layers, and five convolution layers with filter sizes 5 in the second and third layers. Further analysis on the results is given in Parsa et al. (2019a).

For case study two given in Table 3, we show the convenience of changing HP types within the H-PABO framework by incorporating the choice of optimizer as an HP. In Figure 4B,C, H-PABO estimates the Pareto region with 39 and 22 evaluations, respectively. The complexity and predictability of the problem upon changes of HP combinations define the speed of H-PABO in predicting the Pareto region.

### 3.2. Results for SNN

Table 4 shows a summary of the selected ranges for the hyperparameters (HPs) for case studies in SNN domain given in Table 2. In this table, *b*_*k*_, *p*_*k*_, [*c*_*k*_, *C*_*k*_], *function*, and *interval* are from the input encoding module, *population size, mutation rate*, and *crossover rate* are for EONS evolutionary-based training algorithm, and *synaptic weight, neuron threshold*, and *synaptic delay* belong to the underlying neuromorphic hardware. The input encoding hyperparameters include several approaches such as *binning-based*, using *b*_*k*_ as the number of bins required for each input values, *spike-count* with *p*_*k*_ as the maximum number of spikes to encode a single input value, *charge-value* with [*c*_*k*_, *C*_*k*_] on injecting a specific charge to fire a neuron, *function* on how to map the values to spikes, and *interval* to define the interval between pulses. For more details on each of these hyperparameters please refer to Parsa et al. (2019b); Schuman et al. (2019).

**Table 4:** Evaluated parameters for case studies four to nine for SNNs.

**Hyperparameters**	**Case study four**	**Case study five**	**Case studies six, and seven**	**Case studies eight, and nine**
*b*_*k*_	1, 2, 4, 8	2, …, 8	2, 4, 8	2, 4, 8, 10, 12
*p*_*k*_	1, 2, 4, 8	1, …, 12	4, 8	2, 4, 8, 10, 12
[*c*_*k*_, *C*_*k*_]	[0,0.5],[0,1], [0.25,0.5], [0.25,1], [0.5,0.5],[1,1]	[0,0.5],[0,1], [0.25,0.5],[0.25,1], [0.5,0.5],[1,1]	[0,1], [0.5,0.5], [1,1]	[0,0.5],[0,1], [0.25,0.5], [0.25,1], [0.5,0.5],[1,1]
Function	simple, flip-flop, triangale	simple,flip-flop,triangale	simple, flip-flop	simple, flip-flop, triangale
Interval	1	1, …, 5	0, 1	0, 1, 2
Population size	1,000	600, 800, 1,000, 1,200, 1,500, 2000	10, 100, 500	10, 100, 500, 700
Mutation rate	0.9	0.6, 0.7, 0.8, 0.9	0.2, 0.6, 0.9	0.2, 0.6, 0.9
Crossover rate	0.5	0.3, 0.4, 0.5, 0.6, 0.7	0.3, 0.5, 0.9	0.3, 0.5, 0.9
Synaptic weight	[-255,255]	[-127,127],[-255, 255] [-511, 511],[-1023, 1,023]	-	-
Neuron threshold	[0,1,023]	255, 511, 1023	-	-
Synaptic delay	127	15, 31, 63, 127, 255	-	-
Neural Accelerator	DANNA2	DANNA2	mrDANNA	mrDANNA
Application	Pole-balance	Pole-balance	six: IRIS seven: Radio	eight: IRIS nine: Radio
Search Space	240	54,432,000	1458	35,640

We first show the importance of hyperparameter optimization for spiking neuromorphic systems by only focusing on single-objective optimization (performance of the system on the task) problem, where grid search results are already available by Schuman et al. (2019). We then continue with Hierarchical-PABO (H-PABO) results for a three-objective optimization problem (performance, energy, and network size).

**Figure 5:**
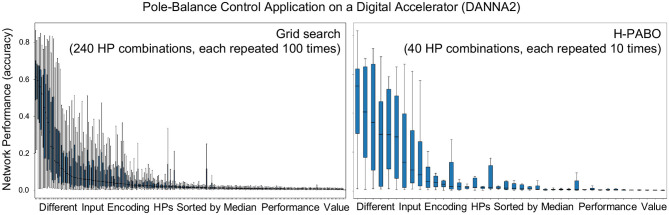
H-PABO results for single-objective hyperparameter optimization (network performance, accuracy only) for case study four in Table 2. Reproduced with permission from (Parsa et al., 2019b).

***Single-Objective Optimization with Hierarchical-PABO (H-PABO)***: While H-PABO is generally aimed for multi-objective problems, it can easily be reduced to a single-objective optimization by setting objective functions to one. This is the case for case study four, where we are only optimizing a single objective function that is the accuracy of the neural network. The details of this case study is given in Table 4. Figure 5 shows box plot figures with interquartile ranges. The grid search result is produced and published by Schuman et al. (2019) and shown in Figure 5A. For each one of the 240 combinations of the hyperparameters, the network accuracy is calculated and evaluated for 100 times. In Figure 5B, we used H-PABO for the same experiment, and with only 40 hyperparameter combinations, each repeated for 10 times, we are able to predict not only the exact optimum set of hyperparameter, but also predict the same trend in the network accuracy changes for different hyperparameter combinations (Parsa et al., 2019b). In this case study the optimum hyperparameter combination leads to median value of 52%.

The hyperparameters are kept exactly similar between case studies four and five in Table 4. However the ranges for each hyperparameter is increased in case study five. Although all hyperparameters are still in reasonable ranges, the search space is drastically increased to over 54 million different hyperparameter combination in case study five. This shows that in real problems where different hyperparameters exist originating from different modules of the system such as input encoding, hardware, or the training algorithm itself, hyperparameter optimization plays vital role in obtaining the maximum performance of the system. We performed H-PABO to define the set of hyperparameter that optimizes network's accuracy and were able to increase the median value of the accuracy to 70.99% compared to 52% in case study four. Please refer to Parsa et al. (2019b) for more details on single-objective hyperparameter optimization on spiking neural networks.

In Table 5, a sensitivity analysis is performed for H-PABO single objective optimization for different classification applications on two different neural accelerators. These experiments show how sensitive is pole-balance control application to the changes of hyperparameters. If we only change few hyperparameters (all in reasonable ranges), the resulting accuracy will change from 52 to 70.99% (comparing experiments 1 and 2 in Table 5). Based on these experiments, RoboNav appears to be less sensitive to changes in hyperparameters and architectures, but more extensive experiments may be required in order to understand the full impact on this particular application.

**Table 5:** Sensitivity analysis for H-PABO single objective optimization.

	**HPs**	**Experiment 1**	**Experiment 2**	**Experiment 3**	**Experiment 4**
Input encoding HPs	*b*_*k*_	2	2	2	2
	*p*_*k*_	8	12	8	8
	Charge	[0, 0.5]	[0, 0.5]	[0, 0.5]	[0, 0.5]
	Function	Flip-flop	Flip-flop	Flip-flop	Flip-flop
	Interval	1	5	1	2
EONS HPs	Population size	1000	1500	400	1000
	Mutation rate	0.9	0.9	0.9	0.9
	Crossover rate	0.5	0.4	0.5	0.7
Accelerator HPs	Synp weight	[−255, 255]	[−127, 127]	[−255, 255]	-
	Neuron threshold	[0, 1023]	[0, 1023]	[0, 1023]	-
	Synp delay	127	255	15	
Neuromorphic System Performance		52%	70.99%	50%	53%
Accelerator		DANNA2	DANNA2	DANNA2	mrDANNA
Application		Pole-Balance	Pole-Balance	RoboNav	RoboNav

***Three-Objective Optimization with Hierarchical-PABO (H-PABO)***: To validate H-PABO technique for multi-objective hyperparameter optimization problems in SNN domain, we focus on classification application with IRIS (Dua and Graff, 2017), and Radio (Eggensperger et al., 2013) dataset on both digital (Mitchell et al., 2018), and mixed-signal memristive (Chakma et al., 2017) neuromorphic devices. The summary of the case studies six to nine, and their corresponding HP ranges are given in Tables 4, 2, respectively.

**Figure 6:**
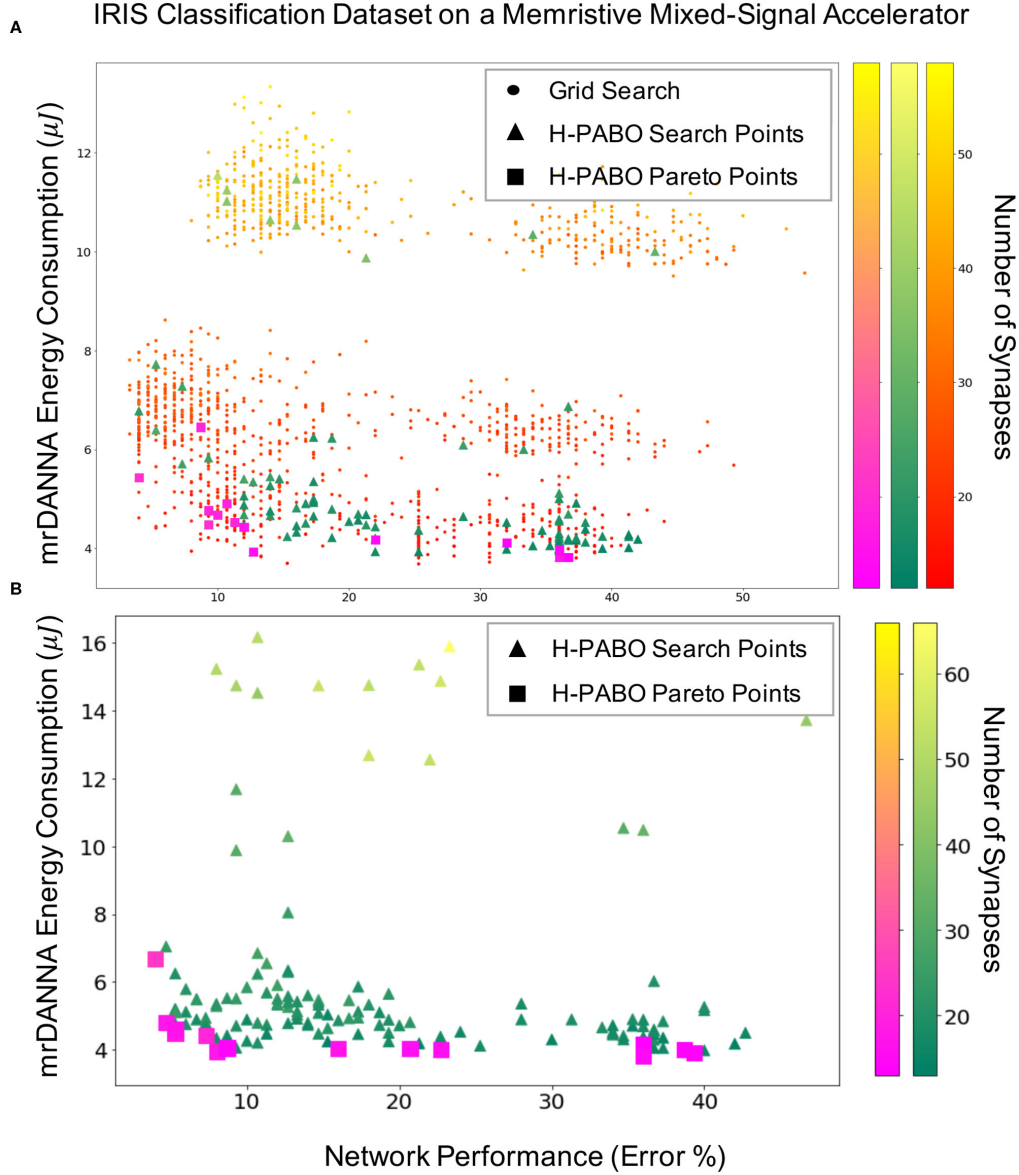
H-PABO results for three-objective hyperparameter optimization (network performance, hardware energy consumption, and number of synapses) for Iris classification dataset on mrDANNA with HP search space of **(A)**, 1458, case study six, **(B)** 35,640, case study eight in Table 4.

Figure 6 demonstrates the Hierarchical-PABO (H-PABO) results in SNN domain on IRIS classification dataset on a mixed-signal underlying hardware [mrDANNA, Chakma et al. (2017)]. Figure 6A shows the H-PABO results compared to grid search for the case study six given in Table 4 with 1458 different sets of HPs. Each point in the three-dimension figure represents network performance, hardware energy consumption, and number of required synapses for a set of HP combination. The number of required synapses increases as the color becomes lighter. The grid search results show that most of the time the energy consumption increases as the number of synapses increase (the top left region of Figure 6A). However, we might also have a larger network with more inhibitory synapses, for example, that would have less activity and thus less energy than a smaller network (top right region). The triangles are the H-PABO search points, and as expected, all different regions of the search space are explored with H-PABO. The H-PABO Pareto points are shown with squares. These points are calculated once the H-PABO search process is completed and are the H-PABO search points that belong to the Pareto frontier. As shown in Figure 6A this calculated Pareto frontier is within close proximity to the actual Pareto frontier of the problem.

Figure 6B shows the H-PABO results for case study eight in Table 4 for the HP search space of 35640 different HP combinations. Once again, we see that all regions of the search space are explored by the H-PABO approach, but that the majority of the H-PABO points are evaluated are in the region of interest and near the H-PABO Pareto front. In this case, H-PABO was able to find well-performing networks with desired characteristics (low energy consumption and relatively few synapses) with significantly fewer evaluates than what would be required for a full grid search of 35,640 points. It is also worth noting that by optimizing over the additional HPs, the H-PABO approach is able to find well-performing networks with better characteristics than the networks found simply optimizing over the smaller set of HPs (shown in Figure 6B).

**Figure 7:**
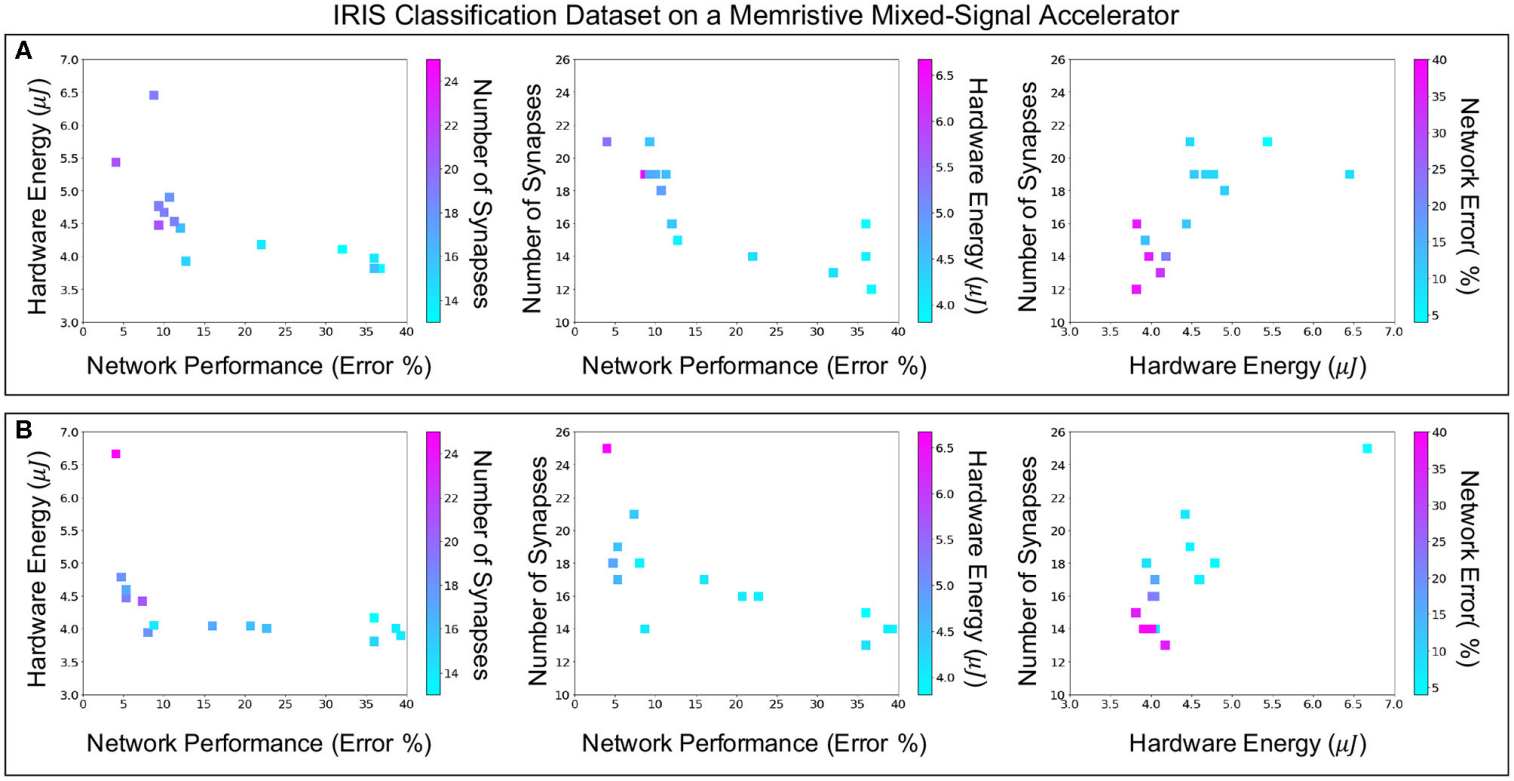
Comparing three-dimensional H-PABO results, pairwise for **(A)** case study six with search space size 1,458, **(B)** case study eight with search space size 35,640.

Figure 7 shows the H-PABO results from Figure 6, but splits the results into three different pairwise comparison plots, for each case study, to show how the different objectives play off of each other. The third objective is also shown in each plot through the color of the squares. With these plots, we can see the different Pareto fronts for each of the pairwise objectives. For example, in the network performance vs. hardware energy plots, we can see that there are trade-offs in energy usage in order to achieve lower error (and similarly for network performance vs. number of synapses). However, the number of synapses and energy usage are relatively correlated, such that fewer synapses typically corresponds to a lower energy value.

**Figure 8:**
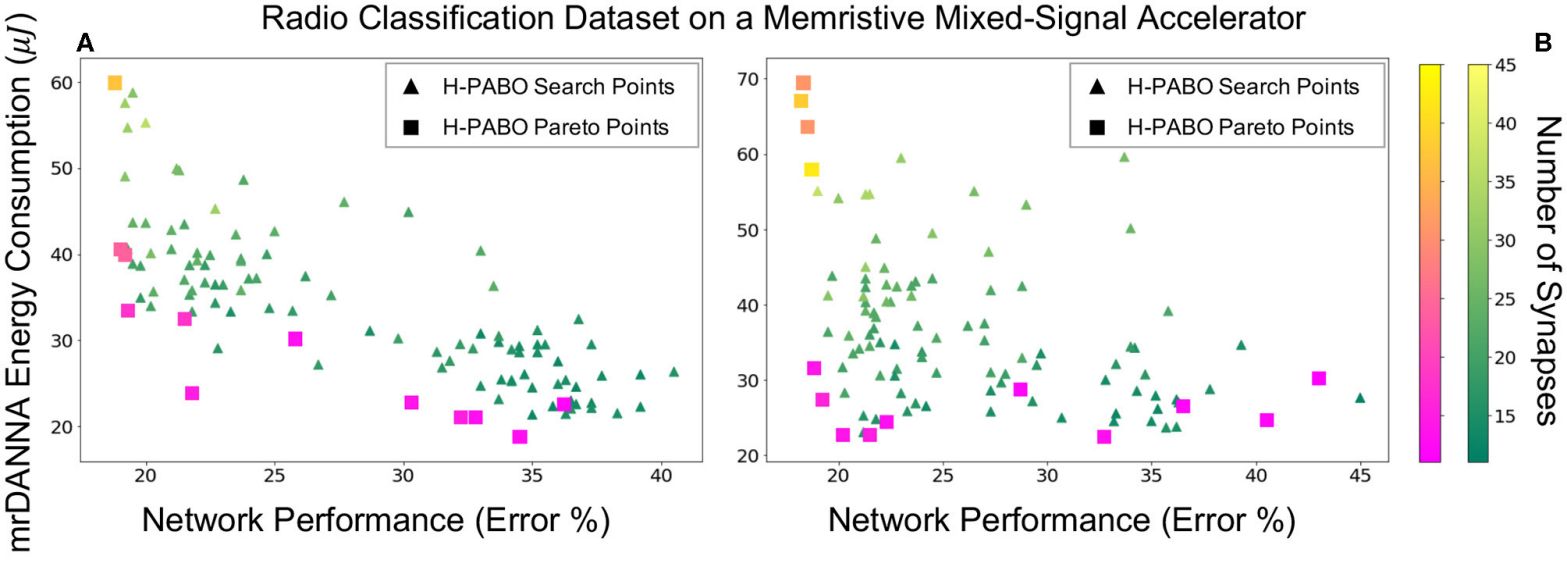
H-PABO results for three-objective hyperparameter optimization (network performance, hardware energy consumption, and number of synapses) for case studies seven and nine in Table 2, Radio classification dataset on mrDANNA, **(A)** search space size 1,458, **(B)** search space size 35,640.

Figure 8 gives the results for case studies seven and nine, in which the H-PABO approach is applied to HP optimization for the Radio classification dataset on the memristive mixed-signal system (mrDANNA). The two case studies look at the same HP combination sets as the Iris dataset and correspond to 1458 and 35640 combinations, respectively. As we can see in the figure, H-PABO once again explores the space of potential solutions but is able to find a Pareto front in relatively few evaluations. Again, similar to the result for the Iris dataset, we can see that by expanding our HP set to the 35640 potential HP combinations, H-PABO is able to achieve overall better performing networks (lower error and energy and fewer synapses required), and in general moving the Pareto front closer to the desired region.

## 4. Discussion and Future Work

In this paper, we propose a novel multi-objective optimization framework based on hierarchical Bayesian optimization and agent-based modeling (Hierarchical-PABO). With its one of a kind structure, and simple yet effective underlying mathematics, we are able to predict a Pareto frontier of a multi-objective hyperparameter optimization for both non-spiking and spiking neural network systems with only few evaluations. This framework paves the way to further analyze and study sensitivity and resiliency of the system due to the changes of the hyperparameters.

The main current limitation of Hierarchical-PABO is scalability and ability to parallelize the approach. The goal of Hierarchical-PABO is predicting the Pareto region for a search space with reasonable ranges for the hyperparameters and with only few evaluations and we do not want to compete with all NAS-based approaches that search the entire search space with massive computational resource requirements. However, improving scalability of Hierarchical-PABO paves the way for incorporating the technique in different frameworks with multiple layers of optimization problems and hyperparameters.

For future work, we intend to fully integrate the Hierarchical-PABO approach into the TENNLab neuromorphic framework by Plank et al. (2018), so that it can seamlessly determine hyperparameters for the neuromorphic framework user. Within that framework, we also intend to apply this hyperparameter framework to other neuromorphic implementations that are supported and other applications, including a variety of control applications (like those described by Plank et al., 2019) and other classification tasks. We also plan to apply H-PABO to determine the hyperparameters for other spiking neural network training approaches, including reservoir computing algorithms, and back-propagation style approaches such as Whetstone (Severa et al., 2019) and SLAYER (Shrestha and Orchard, 2018). To further accelerate the optimization approach, we plan to investigate an implementation of H-PABO for high-performance computers, such as Oak Ridge National Laboratory's Summit supercomputer.

## Data Availability Statement

The following datasets used in this study can be found at:

- Flower17: http://www.robots.ox.ac.uk/~vgg/data/flowers/17/
- CIFAR10: https://www.cs.toronto.edu/~kriz/cifar.html
- IRIS: https://archive.ics.uci.edu/ml/datasets/Iris
- Radio: https://www.deepsig.io/datasets/

All codes for Hierarchical-PABO as well as the simulation codes for pole balance and robotic navigation used in this work are available from the authors on request.

## Author Contributions

MP and KR defined the experimental setup and research experiments for the H-PABO approach for ANN domain, where the extra Bayesian optimizer block in the supervisor agent is off. MP, JM, and CS formulated the experimental setup and research experiments for the H-PABO approach for SNN domain. MP implemented H-PABO and conducted all of the experiments. RP and TP provided feedback and insight into the H-PABO approach for SNN. MP took the lead in writing the manuscript. All authors provided critical feedback and helped shape the research, analysis and manuscript.

## Conflict of Interest

The authors declare that the research was conducted in the absence of any commercial or financial relationships that could be construed as a potential conflict of interest.
