# Supplementary material for: Bayesian Multi-objective Hyperparameter Optimization for Accurate, Fast, and Efficient Neural Network Accelerator Design
Source: Front Neurosci. 2020 Jul 21;14:667. doi: 10.3389/fnins.2020.00667 (PMC7396641; doi:10.3389/fnins.2020.00667)
Supplement: Supplementary file 1 [file Data_Sheet_1.PDF]

Supplementary Material

1 PSEUDO-CODE FOR HIERARCHICAL-PABO

Algorithm 1 illustrates the pseudo-code of H-PABO framework. In this triple-objective optimization algorithm the black-box objective functions are shown with f_{perf}, f_{eng}, and f_{size}. f_{perf} is the performance of the neural network (ie. error), f_{eng} is the energy consumption of the underlying neural accelerator, and f_{size} is a proxy for area requirement of the design.

In iteration n, we have observations from the isolated Bayesian estimators for the objective functions. Among all these observations, we select and store those points that belong to the Pareto frontier (ie. the HP points coming from any of the D_{perf}, D_{eng}, or D_{size} that are non-dominated), and their corresponding score vector (ie. The vector containing the results of evaluating performance, energy, and size for that specific HP). Please note that this vector is not limited in size and can be adjusted based on the number of objective functions. These non-dominated points for this iteration, create D_{IntPar} set. In this step, assume that you would like to estimate a completely new function using Bayesian optimization (intermediate Pareto function). Bayesian optimization helps in estimating black-box functions with sets of observations. In the second level, this black-box function is a intermediate function that changes in every iteration as we learn more about the isolated Bayesian estimators in the first level. To build a posterior model for this intermediate function, we require likelihood model (ie. our observations) and a prior model. Observations are non-dominated HPs stored in the D_{IntPar} set. The prior Gaussian distribution model uses these observations along with a score dedicated for each observation. In H-PABO we use a normalized summation of the score vectors for each HP, and in this way, we represent a single score for each non-dominated point. We estimate a Gaussian distribution for these non-dominated HPs (From D_{IntPar}) and their corresponding scores IntPar_n, calculate an acquisition function ($AF_n(\widetilde{\text{IntPar}})$, and optimize it. The optimum point of this acquisition function is the new HP that helps moving the current Pareto to the corner. This new HP is then added to all isolated Bayesian estimators in the first level and help with improving those estimations. By repeating this process, we move the intermediate function in the second level closer to the corner and therefore actual Pareto region of the problem.

In H-PABO framework, there are two different stopping criteria. One is after a predefined number of iterations in the H-PABO process, and the other one is when the new observations (new set of hyperparameter) does not improve the Bayesian estimation. This happens when the surrogate model (acquisition function) converges to zero and the optimum point of this acquisition function cannot suggest a new set of hyperparameter that helps in exploring and exploiting the search space.

2 DETAILS OF ARCHITECTURES USED IN EXPERIMENTAL SETUP FOR ANN

Throughout this supplementary document we present artificial neural network (ANN) architecture with the following notation: $-$ for dividing layers, c for convolution layers, p for pooling layers, and fc for fully connected layers. For example $128 \times 128 \times 3 - 12c5 - 2p - 10o$ is a four-layer ANN with $128 \times 128 \times 2$ input followed by 12 convolution filters with size 5, a 2×2 pooling layer, and finally 10 output neurons. Details of the AlexNet and VGG19 architectures for Flower17 and CIFAR10 dataset are given in Table S1, respectively. This table only shows a sample architecture for AlexNet, and VGG19, and the architectures are modified based on the hyperparameters given in the experimental setup for ANN.

Algorithm 1 Hierarchical-PABO (for triple-objective optimization: performance, energy, size)

Notations: AF: Acquisition Function; p-norm: $||.||_p$; HP: hyperparameter;
n: iteration number; O: Observations; β: estimated Pareto front set; $I_K := \{1, 2, ..., K\}$

Inputs: Three objective functions (performance, energy and size): $f_{\text{perf}}, f_{\text{eng}}, f_{\text{size}}$
HP_{all}: The set containing all possible combinations of hyperparameters (HPs)
Initial training datasets: θ, Γ, Ψ

Initialize: $n \leftarrow 1$
$flag \leftarrow True$
$\theta_n = \Gamma_n = \Psi_n = \{hp1, hp2\}$, where set $\{hp1, hp2\}$ is randomly selected from HP_{all}
$O_n(\theta) \equiv [f_{\text{perf}(\theta_n)}, f_{\text{eng}(\theta_n)}, f_{\text{size}(\theta_n)}]$
$D_{\text{perf}} = \emptyset$: Set for storing all selected HPs for estimating f_{perf}
$D_{\text{eng}} = \emptyset$: Set for storing all selected HPs for estimating f_{eng}
$D_{\text{size}} = \emptyset$: Set for storing all selected HPs for estimating f_{size}
$D_{\text{IntPar}} = \emptyset$: Set for storing all selected HPs for estimating IntPar (intermediate Pareto front)

============================= **LEVEL 1** ====================================

1: $D_{\text{perf}} = D_{\text{perf}} \cup \theta_n, \quad D_{\text{eng}} = D_{\text{eng}} \cup \Gamma_n, \quad D_{\text{size}} = D_{\text{size}} \cup \Psi_n.$

2: Posterior Gaussian distributions:
$\tilde{f}_{\text{perf}} = p(\tilde{f}_{\text{perf}}|(f_{\text{perf}}, D_{\text{perf}})), \quad \widetilde{f_{\text{eng}}} = p(\tilde{f}_{\text{eng}}|(f_{\text{eng}}, D_{\text{eng}})), \quad \tilde{f}_{\text{size}} = p(\tilde{f}_{\text{size}}|(f_{\text{size}}, D_{\text{size}}))$

3: while $flag$ do

4: Calculate $AF_n(\tilde{f}_{\text{perf}}), \ AF_n(\tilde{f}_{\text{eng}}), \ AF_n(\tilde{f}_{\text{size}})$

5: $\theta_{n+1} = \underset{HP_{\text{all}}}{\text{argmax}} AF_n(\tilde{f}_{\text{perf}}), \ \Gamma_{n+1} = \underset{HP_{\text{all}}}{\text{argmax}} AF_n(\tilde{f}_{\text{eng}}), \ \Psi_{n+1} = \underset{HP_{\text{all}}}{\text{argmax}} AF_n(\tilde{f}_{\text{size}})$

6: if $\theta_{n+1} = \theta_n$, and $\Gamma_{n+1} = \Gamma_n$, and $\Psi_{n+1} = \Psi_n$:
$flag \leftarrow False$

7: else:

8: $D_{\text{perf}} = D_{\text{perf}} \cup \theta_{n+1}, \quad D_{\text{eng}} = D_{\text{eng}} \cup \Gamma_{n+1}, \quad D_{\text{size}} = D_{\text{size}} \cup \Psi_{n+1}.$

9: Evaluate $O_{n+1}(\theta)$, $O_{n+1}(\Gamma)$, $O_{n+1}(\Psi)$

============================= **LEVEL 2** ====================================

10: calculate $\beta_n = \{\forall\, i \in I_K | \quad \beta_n^i\}$
(where $O_n(\beta)$ are non-dominant points. Please note K maybe different in each iteration)

11: $D_{\text{IntPar}} = D_{\text{IntPar}} \cup \beta_n$

12: calculate $O_{n,norm}(\beta)$, (by normalizing each element of $O_n(\beta)$ to $[0, 1]$)

13: $\text{IntPar}_n = \{\forall\, i \in I_K | \quad \text{IntPar}_n^i = ||O_{n,norm}^i||_1\}$

14: $\tilde{\text{IntPar}}_n = p(\tilde{\text{IntPar}}_n|(\text{IntPar}, D_{\text{IntPar}}))$

15: Calculate $AF_n(\tilde{\text{IntPar}})$

16: $\beta_{n+1} = \underset{HP_{\text{all}}}{\text{argmax}} AF_n(\tilde{\text{IntPar}})$ (Next best data set to move the current Pareto the corner)

17: $D_{\text{perf}} = D_{\text{perf}} \cup \beta_{n+1}, \quad D_{\text{eng}} = D_{\text{eng}} \cup \beta_{n+1}, \quad D_{\text{size}} = D_{\text{size}} \cup \beta_{n+1}.$

18: Evaluate $O_{n+1}(\beta)$

19: $n \leftarrow n + 1$

20: update $\tilde{f}_{\text{perf}}, \tilde{f}_{\text{eng}}, \tilde{f}_{\text{size}}, \tilde{\text{IntPar}}$.

Table S1. Details of Architectures used in Experimental Setup for ANN section

Name	Architecture
AlexNet	$227 \times 227 \times 3 - 96c5 - p3 - 256c3 - p3 - 384c3 - 384c3 - 256c3 - p3 - 4096fc$ $-4096fc - 17fc$
VGG19	$32 \times 32 \times 2 - 64c3 - 64c3 - p2 - 128c3 - 128c3 - p2 - 256c3 - 256c3 - 256c3$ $-256c3 - p2 - 512c3 - 512c3 - 512c3 - 512c3 - p2 - 4096fc - 4096fc - 1000fc$
